# Clinical Efficacy of the HIV Protease Inhibitor Indinavir in Combination with Chemotherapy for Advanced Classic Kaposi Sarcoma Treatment: A Single-Arm, Phase II Trial in the Elderly

**DOI:** 10.1158/2767-9764.CRC-24-0102

**Published:** 2024-08-15

**Authors:** Cecilia Sgadari, Biancamaria Scoppio, Orietta Picconi, Antonella Tripiciano, Francesca Maria Gaiani, Vittorio Francavilla, Angela Arancio, Massimo Campagna, Clelia Palladino, Sonia Moretti, Paolo Monini, Lucia Brambilla, Barbara Ensoli

**Affiliations:** 1 National HIV/AIDS Research Center, Istituto Superiore di Sanità, Rome, Italy.; 2 Dermatology Unit, Fondazione IRCCS Cà Granda Ospedale Maggiore Policlinico, Milan, Italy.

## Abstract

Kaposi sarcoma is a rare angioproliferative disease associated with human herpes virus-8 (HHV-8) infection. Kaposi sarcoma is frequent and aggressive in HIV-infected people, whereas the classic form (CKS) generally has an indolent course. Notably, all conventional therapies against Kaposi sarcoma have only temporary efficacy. We have previously shown that indinavir, a HIV protease-inhibitor with direct antiangiogenic and antitumor activity, is safe and effective in patients with early CKS, whereas effects are less prominent in advanced disease, probably due to the larger tumor mass. Therefore, the clinical response to indinavir was assessed in patients with advanced CKS after debulking chemotherapy. This was a monocentric phase 2 trial in elderly with progressive/advanced CKS treated with debulking chemotherapy and indinavir combined, followed by a maintenance phase with indinavir alone. Secondary endpoints included safety and Kaposi sarcoma biomarker evaluation.

All evaluable patients (22) responded to debulking therapy. Out of these, 16 entered the indinavir maintenance phase. The overall response rate at end of maintenance was 75% (estimated median response-duration 43 months). Moreover, most responders showed further clinical improvements (lesion number/nodularity) during maintenance and post-treatment follow-up. Notably, after relapse, progressors did not require systemic Kaposi sarcoma therapy and showed clinical improvements (including disease stabilization) remaining on study. Responders also showed immune status amelioration with a consistent B-cell increase and positive changes of other biomarkers, including anti-HHV-8 natural killer activity. In advanced CKS a strategy combining indinavir and chemotherapy is safe and associated with high and durable response rates and it could be rapidly adopted for the clinical management of these patients.

**Significance:**

This phase-2 trial showed that the HIV protease inhibitor indinavir may boost and extend the duration of the effects of chemotherapy in elderly with advanced progressive classic Kaposi sarcoma, without additional toxicity. Further, the amelioration of the immune status seen in responders suggests a better control of HHV-8 infection and tumor-cell killing. Thus, indinavir combined with chemotherapy may represent an important tool for the clinical management of classic Kaposi sarcoma in elderly patients.

## Introduction

Kaposi sarcoma is an angioproliferative tumor classified as a rare disease characterized by the proliferation of “spindle-like” cells (Kaposi sarcoma cells), a heterogeneous cell-population expressing markers of activated vascular and lymphatic endothelial cells (EC; ref. 1). Kaposi sarcoma is found as different clinical-epidemiological forms occurring in elderly men of the Mediterranean area [classic Kaposi sarcoma (CKS)], sub-Saharan Africans (endemic or African Kaposi sarcoma), organ transplant recipients (post-transplant Kaposi sarcoma), and HIV-infected subjects (AIDS-Kaposi sarcoma; ref. 2). Independent of these different forms, the histopathology of Kaposi sarcoma is always the same. In the early stage, lesions resemble a granulation tissue, while in the advanced stage, lesions appear as a frank tumor. All forms of Kaposi sarcoma are invariably associated with infection by human herpesvirus-8 (HHV-8), which represents a necessary, although not sufficient, etiological factor for Kaposi sarcoma development (1, 2).

Kaposi sarcoma generally first appears with single or multiple independent macular skin lesions in the context of a dysregulation of the immune system characterized by activation of CD8^+^ T cells and increased production of inflammatory cytokines (1). In turn, these inflammatory cytokines induce the recruitment of circulating inflammatory cells and EC into tissues, induce the production of angiogenic factors such as fibroblast growth factor-2 (FGF-2) and vascular endothelial growth factor (VEGF), activate EC to acquire the phenotype of Kaposi sarcoma cells, and reactivate HHV-8 (1). Over time, the early-Kaposi sarcoma reactive angiogenic/inflammatory lesions can progress toward a true tumor, with nodular lesions that may coalesce into large tumors. However, all stages of Kaposi sarcoma are characterized by an increased activity of matrix metalloproteases (MMP), enzymes that, by degrading the extracellular matrix and the basement membrane, promote angiogenesis as well as the invasion of tissues by neoplastic cells. Tumor progression is likely due to the deregulated expression of oncogenes and oncosuppressor genes and due to the effects of proliferative and antiapoptotic viral genes, including HHV-8 latency genes and, in AIDS-Kaposi sarcoma, the HIV-1 Tat protein (3). In addition, immune evasion mechanisms, immune defects, or overt immune suppression also play a role in Kaposi sarcoma by allowing uncontrolled HHV-8 replication or tumor growth.

Despite recent improvements, Kaposi sarcoma continues to be an incurable disease in all epidemiologic settings (4, 5). Patients with limited, early-stage Kaposi sarcoma are generally treated by removal or topical/intralesional therapy, whereas rapidly progressing or visceral Kaposi sarcoma with life-threating lesions requires systemic antiproliferative treatments. Current therapeutics, however, have only temporary efficacy and the disease generally reoccurs and progresses, seriously hampering patients’ quality of life. Of note, the advent of effective antiretroviral therapy has led to a reduced incidence and/or regression of HIV-associated tumors, particularly Kaposi sarcoma (6). Although HIV suppression and the consequent restoration of the immune response by antiretroviral therapy certainly play a key role in HIV-associated tumors, evidence indicates that anti-HIV drugs, including HIV-1 protease inhibitors (HIV-PI), have direct antitumor activities that are beyond effects on immune reconstitution obtained by suppressing HIV replication. In fact, HIV-PIs exert direct antiangiogenic, anti-inflammatory, and antitumor actions that are unrelated to their antiretroviral activity (6). In particular, HIV-PIs impair EC, Kaposi sarcoma, and tumor invasion via a block of activation of MMPs. As a consequence, HIV-PIs block angiogenesis and tumor-cell invasion in HIV-free *in vivo* models of Kaposi sarcoma and of other highly prevalent human tumors (6–11). In addition, by targeting the MMP-9/VEGF proangiogenic axis, HIV-PIs also promote normalization of both vessel architecture and functionality and enhance the delivery and antitumor activity of conventional chemotherapy in a preclinical model of cervical cancer (9).

Based on these studies, we conducted a proof-of-concept phase II trial for the evaluation of the efficacy of the HIV protease inhibitor indinavir in early- and late-stage CKS. Indinavir treatment was safe and led to a clinical response in 61.5% of the participants (12). The response rate was higher in patients with early-stage Kaposi sarcoma (9/12, 75%) as compared to subjects with late-stage Kaposi sarcoma (7/14, 50%). Disease relapse occurred after a median of 19 weeks and was significantly associated with late-stage Kaposi sarcoma (*P* = 0.0350; Fisher’s exact test). In addition, a favorable clinical course was associated with high drug plasma levels, reduced production of FGF-2, lower number of circulating EC (CEC), and a decrease in antibody titers against HHV-8 (12). However, indinavir had little or no effect on advanced CKS characterized by coalescent lesions forming large tumor masses, particularly when complicated by lymphedema, lymphorrhea, ulcerations, and/or bacterial infections. Therefore, these data suggested that indinavir could be more effective in patients with advanced CKS only upon debulking therapy.

Here we report the results of a clinical study aimed at evaluating the effects of a debulking chemotherapy combined with indinavir followed by a maintenance phase with indinavir alone in elderly patients with advanced progressive CKS. Although presenting with concomitant diseases, patients tolerated treatment well and, after the debulking therapy, experienced further improvement of the clinical response and its duration, as well as improvement of the immune status and of biological markers associated with Kaposi sarcoma development and progression. Thus, indinavir boosts and maintains the effect of chemotherapy against advanced progressive CKS in elderly patients.

## Materials and Methods

### Study design and participants

A proof-of-concept, single-arm, phase 2 trial was conducted in patients with advanced progressive CKS to determine the clinical response to daily indinavir oral administration in combination with a debulking chemotherapy based on cycles of systemic vinblastine + bleomycin (induction phase). This was followed by a maintenance phase with indinavir alone and a post-therapy follow-up. The study protocol was later amended to allow the use of vinblastine alone during the debulking phase in patients with a previous bleomycin total load higher than allowed (see below), resistant or relapsed Kaposi sarcoma, or a negative clinician opinion for bleomycin use.

This single-center study was conducted at the Dermatology Unit, Fondazione IRCCS Cà Granda Ospedale Maggiore Policlinico, Milan, Italy, from June 2008 (first participant enrolled) to October 2015 (last study visit) after approval from the competent Ethics Committees (ISS and participating clinical center) and regulatory authority (General Director of the participant hospital). The study was open to male and female adult patients (aged ≥18 years) with histologically proven advanced CKS (stage III or IV; ref. 13). The key exclusion criteria were history of HIV, concomitant illness and neoplastic diseases, severe broncho-pneumopathies, severe nephropathies or nephrolithiasis in the last 5 years, and laboratory-parameter abnormalities, including hemoglobin < 10.0 g/dL, platelet count < 100,000/mm^3^, neutrophil count < 1,500/mm^3^, serum creatinine > 1.2 mg/dL, creatinine clearance > 100 mL/min ±25, or hepatic enzymes/total bilirubin > 3-fold upper limit of normal.

The study was conducted in accordance with the Declaration of Helsinki and the International Conference on Harmonization Good Clinical Practice guidelines. All patients provided written informed consent before study entry. The trial was registered with the EudraCT, 2007-000567-26 and ClinicalTrials.gov, NCT01067690.

The number of patients needed for the study was 25. Sample size was initially calculated with the Simon’s minimax two-stage design, based on responses obtained at the clinical site with vinblastine and bleomycin combined. After the study was amended to allow the use of vinblastine alone during induction, and in consideration of the low incidence of CKS, the same sample size was maintained.

### Procedures and outcome measures

To assess tolerance, patients initially started intravenous vinblastine with a dose escalation of 4, 6, and 8 mg weekly, in association with daily indinavir (800 mg × 2/die, orally). If well tolerated, after a 3-week stop, patients received vinblastine 10 mg i.v. ± bleomycin 15 mg i.m., every 3 weeks, in association with daily indinavir. The most appropriate debulking treatment was decided by the investigator, based on patient’s clinical evaluation and previous bleomycin load. The allowed total bleomycin dose was 220 mg/patient (including previous treatments). Patients received two additional cycles of vinblastine ± bleomycin as consolidation after achieving the maximal clinical response, defined as complete remission or no further reduction of tumor mass or Kaposi sarcoma-associated complications. Nonresponding patients were shifted to second-line chemotherapy (cycles of paclitaxel, 100 mg i.v. once/week, followed by two additional cycles as consolidation), with continuous indinavir treatment. This phase was followed by a 12-month maintenance treatment with indinavir alone scaled up to 800 mg × 3/die orally and by a post-therapy follow-up of 12 months. Dose and schedule modifications were specified for hematologic and other ≥grade 3 toxicities.

Indinavir (Crixivan) was supplied by Merck Sharp & Dohme Italia. Vinblastine, bleomycin, and paclitaxel were supplied by the hospital pharmacy.

Tumor assessment and staging were performed at baseline by evaluation of total Kaposi sarcoma lesion count and nodularity, size and nodularity of five marker skin lesions, presence and extension of Kaposi sarcoma-associated lymphedema, and mucosal and visceral involvement evidenced by chest X-ray, otorinolaryngological and gynecological evaluation, esophagus–gastro-duodenum endoscopy, ultrasound examination of abdominal cavity, and colonoscopy for participants with positive fecal occult blood test (14). Tumor assessment was repeated at month 3 and 5 during induction and then every 2 months during maintenance and post-therapy follow-up. Documentation of mucosal and visceral involvement was repeated at study exit only in those participants who resulted positive at screening.

A clinical evaluation and laboratory monitoring (including assessment of performance status according the Karnofsky’s index, and blood and urine sampling) and adverse event (AE) evaluation were done at screening, during induction before each chemotherapy cycle and every 2 months during the maintenance phase. In patients receiving bleomycin, spirometry, measure of gas transfer, and blood oxygen saturation was also performed at screening and at month 4 during induction.

The primary endpoint was the investigator-assessed overall clinical response rate, defined by the occurrence of clinical complete response (CR), partial response (PR), or improved disease (ID) recorded from the start of treatment upon the induction phase (debulking chemotherapy) and at treatment completion.

Clinical response was assessed by following the modified AIDS Clinical Trial Group criteria, as previously used in patients with HIV or non-HIV Kaposi sarcomas with some modifications (14–16). Briefly, a CR required clinical resolution of all lesions and tumor-associated edema. A PR required a ≥50% decrease in lesion number, size of the five indicator lesions, or flattening of at least 50% of previously raised lesions, with resolution or improvement of tumor-associated edema, and not meeting criteria for progressive disease (PD). ID required <50% decrease of lesion number, flattening of <50% of raised lesions, or <50% decrease of size of the indicator lesions, with persistence, but not appearance or worsening, of tumor-associated edema, and not meeting criteria for PD. Responses had to be sustained for at least 4 weeks. Stable disease (SD) was defined as any response not meeting the criteria for CR, PR, ID, or PD. As it is recognized that patients may occasionally show a reduction in the size and/or number of lesions at the same time of the appearance of few new lesions that can regress in few weeks (14), in this study a PD was defined as ≥25% increase in the total lesion count, with >3 new infiltrated lesions (nodules/plaques), persisting at the following assessment, or a ≥25% increase in the size of existing lesions, or a change from macular to plaque-like or nodular of ≥25% lesions.

The secondary endpoints were the duration of response (recorded from the start of treatment) and the time to progression (defined as the time from start of treatment to first progression) for the participants who entered the maintenance phase. If a patient dropped out before the study completion, clinical response was censored at the time of the last disease assessment. Other endpoints included safety, immunological status, and key biological markers important in Kaposi sarcoma pathogenesis.

### Biological endpoints evaluation

#### Quantification of plasma MMP-2 levels

The commercially available MMP-2 activity assay system (Biotrak, GE Healthcare) was used for the quantitative determination of active MMP-2 levels in heparin-collected plasma samples, according to the manufacturer’s instructions. The assay can measure active MMP-2 in a range of 0.19 to 3 ng/mL with a sensitivity of 190 pg/mL.

#### Determination of CEC and endothelial progenitor cells

CEC and endothelial progenitor cell (EPC) counts were determined by four-color flow cytometry, as previously described (17). Briefly, peripheral blood leukocytes, isolated from the whole blood, were stained with a panel of monoclonal antibodies directed against CD45 (Becton Dickinson Biosciences, San Jose, CA USA), CD133/1 (AC133 epitope, Miltenyi Biotec, Bergisch Gladbach, Germany), CD31, and CD146/P1H12 (Becton Dickinson Biosciences, San Jose, CA USA), evaluated by FACSCanto flow cytometer and analyzed with the BD FACSDiva Software (BD Biosciences) upon acquisition of 10^6^ cells per sample. CEC and EPC were quantified as the percentage of CD45^−^/CD31^+^/CD146^+^ and CD45^−^/CD31^+^/CD133^+^ cells, respectively. The absolute number of CEC and EPC was calculated from the absolute number of white blood cells provided by a hematology analyzer and the percentage of CEC and EPC (17).

#### Lymphocyte phenotyping

Lymphocyte subsets phenotype [CD4^+^ and CD8^+^ T cells, natural killer (NK), and B cells] were performed by BD Multitest six-color TBNK reagent with BD Truecount tubes, (BD Biosciences) on whole blood. Samples were processed by FACSCanto flow cytometer (BD Biosciences) and data were analyzed by FACSDiva clinical software.

#### NK cell–mediated cytotoxicity activity

To assess the NK cytotoxic activity, human peripheral blood mononuclear cells, K562 cells, or BCBL1 cells (a primary effusion lymphoma-derived cell line, which is infected with HHV-8) were used as target cells. A commercially available cell-mediated cytotoxicity kit [LIVE/DEAD Cell-Mediated Cytotoxicity Kit (L7010), Molecular Probes, Invitrogen] was used according to the manufacturer’s instructions. Samples were analyzed by flow cytometry in the FL1 and FL3 channels for DiOC_18_ and PI, respectively, to determine the percentage of dead target cells. Analysis was performed with FACSCanto using DIVA clinical software (Becton Dickinson).

### Statistical analysis

Clinical response was evaluated per protocol in all enrolled patients who underwent at least a clinical evaluation after starting treatment. Safety population included all enrolled participants who started therapy. Descriptive statistics by Common Terminology Criteria for AEs grading of the National Cancer Institute (version 3.0) was used in all safety analysis (laboratory and clinical AE) assessments.

Continuous data were summarized using descriptive statistics using *N*, median, interquartile range (IQR). Categorical data were presented using *N* and %.

The Pearson χ^2^ test was performed to compare categorical data between groups. The Wilcoxon signed rank-sum test for paired data was used to analyze changes from enrolment and from baseline of maintenance within responders and progressors, while the Mann–Whitney test was used to evaluate differences between responders and progressors at each timepoint and between treatment groups for biological and immunological biomarkers.

The Kaplan–Meyer product-limit method was used to estimate the duration of response and the time to progression; 95% confidence interval (CI) for these parameters were calculated with the same method.

Statistical analyses were carried out at two-sided with a 0.05 significance level, using SAS (Version 9.4, SAS Institute Inc.) and STATA (version 8.2, StataCorp LLC).

### Data availability

Individual participants’ data are protected for confidentiality reasons and can be made available only upon approval of a collaborative research proposal by the corresponding author, the ISS Ethical Committee, and the ISS Data Protection Officer. The research proposal must adhere to the Informed Consent signed by the participants.

## Results

**Figure 1:**
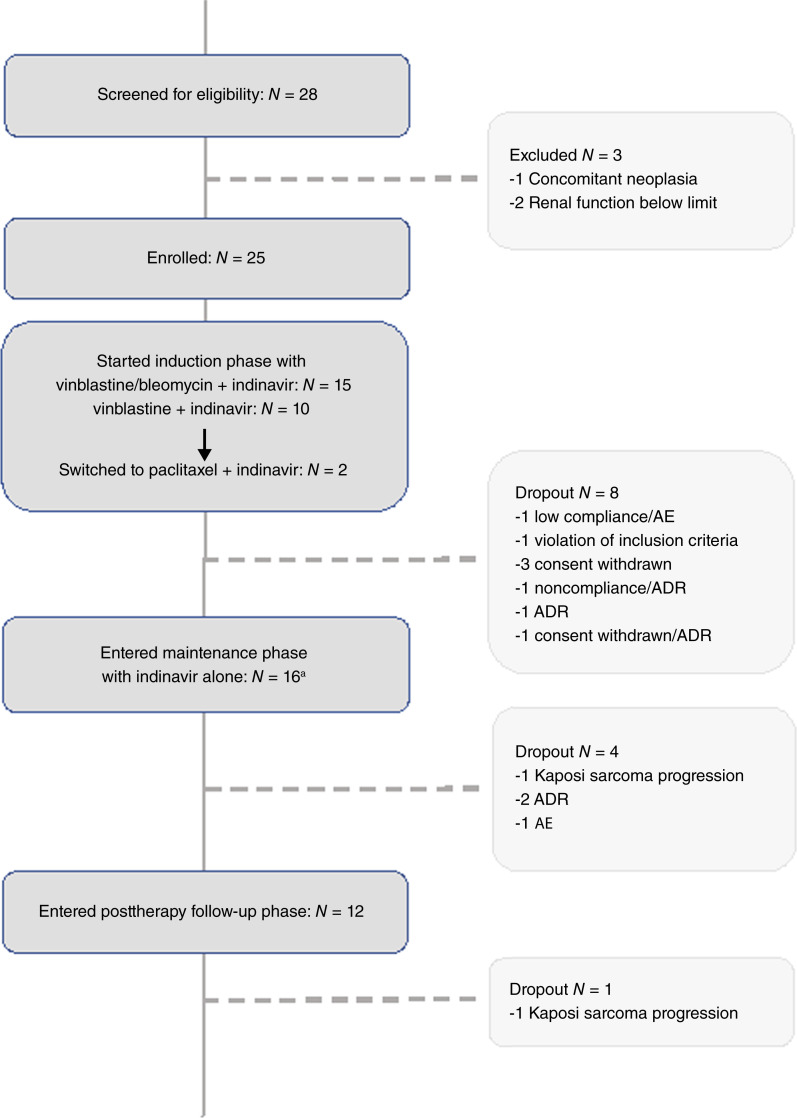
Trial flow chart. The diagram shows the number of patients screened for eligibility (with exclusion reasons), and the number of participants enrolled and completing the induction, maintenance and follow-up phases (with dropout reasons). “a” Denotes one participant dropped out before starting maintenance.

Twenty-eight patients with advanced CKS were screened for eligibility and 25 participants were enrolled and started therapy (Fig. 1). Baseline characteristics are summarized in Table 1. Twenty participants (80%) were male, with a median age of 68 years (IQR: 11). Most patients (n. 23, 92%) had a progressive disease (stage B), with several body sites involved (median *n* = 4), and a high number of skin lesions (median *n* = 48) with an infiltrating behavior (median numbers of nodules *n* = 4, median numbers of plaques *n* = 7), as expected in advanced Kaposi sarcoma. Three participants presented with mucosal/visceral Kaposi sarcoma lesions (one oral, one esophageal, one gastric) and 15 (60%) with lymphedema. No other Kaposi sarcoma-associated complications or symptoms were observed and the participants’ performance status was good (score 100%). The median time from Kaposi sarcoma diagnosis was 6 years. Most patients (n. 19; 76%) had previously received at least one anti-Kaposi sarcoma therapy, including local treatment (16 participants) and/or systemic chemotherapy (seven participants).

**Table 1:** Baseline characteristics of study participants

	Total (*N* = 25)
Age, median years (IQR)	68 (11)
Sex, *n* (%)	
Male	20 (80)
Female	5 (20)
Kaposi sarcoma stage, *n* (%)	
IIIA	2 (8)
IIIB	19 (76)
IVB	4 (16)
Kaposi sarcoma lesions	
Body sites involved, Median (IQR)	4 (3)
Number of lesions, Median (IQR)	48 (19)
Mucosal/Visceral involvement, *n* (%)	3 (12)
Kaposi sarcoma–associated lymphedema, *n* (%)	15 (60)
Kaposi sarcoma diagnosis, median years (IQR)	6 (9)
Previous Kaposi sarcoma treatments, *n* (%)	
None	6 (24)
Local treatments	16 (24)
Chemotherapy	7 (28)
Radiotherapy	3 (12)
Concomitant (active) diseases, *n* (%)	20 (80)
Concomitant medications, *n* (%)	18 (72)

Data are shown as absolute number and percentage (%).

Given their advanced age, most participants (n. 20, 80%) presented with one or more concomitant pathologies. Cardiovascular, metabolic, urological, and gastrointestinal conditions were the most represented among the active concurrent illnesses, which required a pharmacological intervention in 18 participants (Table 1; Supplementary Table S1).

Of the 25 participants who entered the induction phase, 15 received a debulking therapy based on both vinblastine and bleomycin (median cycle n. 8; IQR 2), and 10 received vinblastine alone (median cycle n. 11; IQR 2), all associated with indinavir (Fig. 1). There were no significant differences at baseline between the two treatment groups in terms of years from Kaposi sarcoma diagnosis, Kaposi sarcoma stage at baseline, or total lesion number, except for the median number of body sites with lesions, which was higher in the vinblastine + bleomycin group (Mann–Whitney test; *P* = 0.0032). As foreseen by the study protocol, two patients switched to a paclitaxel-based chemotherapy after failing to respond to vinblastine or vinblastine/bleomycin (two patients; cycle n. received: 15 and 12, respectively; Fig. 1). A total of 17 patients completed the debulking phase [(one dropped out for violation of inclusion criteria, three for consent withdrawal, one for consent withdrawal and adverse drug reaction (ADR), one for ADR, two for low compliance and AE or ADR)] (Fig. 1).

As per protocol, the clinical response to debulking therapy based on vinblastine ± bleomycin or paclitaxel combined with indinavir was evaluated in 22 patients, as three participants dropped out very early after therapy initiation (two patients during the vinblastine escalation phase and one right after the first vinblastine/bleomycin cycle) before performing a disease assessment. The median follow-up of evaluable patients was 35 months (IQR 7). All evaluable patients (22; 100%) showed a response to the induction phase. In detail, one patient obtained a CR and 16 a PR. A lesion improvement (less than 50% clinical response) was also observed in the five evaluable patients who dropped out before completing the debulking therapy (Table 2). Of note, these responses were documented to be sustained for at least 4 weeks as per clinical response assessment in two participants. The disease control rate (CR + PR + ID) upon induction was 100%. The response rate between the participants that received vinblastine or vinblastine + bleomycin chemotherapy combined with indinavir was not statistically different. Among the responders, the median time to response for chemotherapy and indinavir combined was 6 months (IQR 3), which is in line with what was reported by the same clinical center for vinblastine + bleomycin or vinblastine alone [6 months (IQR 8) or 7 months (IQR 4), respectively] (18, 19).

**Table 2:** Clinical responses upon induction (6–8 months), maintenance (12 months), and post-therapy follow-up (12 months)

Study phase	Total *N*	Disease control rate	CR *n* (%)	PR *n* (%)	ID *n* (%)	SD *n* (%)	PD *n* (%)
Response upon induction	22^a^	100%	1 (5%)	16 (73%)	5 (23%)	0	0
Response upon maintenance	16	75%	3 (19%)	9 (56%)	0	0	4 (25%)^b^
Post-therapy follow-up	12^c^	59%	2 (17%)	5 (42%)	0	0	5 (42%)^d^

Data are shown as absolute number (*n*) and percentage (%).

Disease control rate was defined as CR + PR + ID.

a Out of the 25 patients who begun the induction phase, three dropped out very early, before performing a disease assessment, and, as per protocol, were not evaluated for response.

b Out the four patients who progressed during maintenance with indinavir alone, one dropped out and three entered the post-therapy follow-up.

c Four patients dropped-out during maintenance and did not enter the post-therapy follow-up phase (one PD, two ADR, one AE).

d Two participants included among the responders upon maintenance with indinavir alone progressed at month 10 of post-therapy follow-up.

Out of the 17 patients who completed the debulking phase, one patient in PR discontinued for an AE not related to treatment before starting maintenance with indinavir alone.

In the 16 participants who concluded the maintenance phase with indinavir alone, the overall response rate was 75% (12/16; Table 2). In particular, five participants showed a further improvement in lesion number/nodularity with two of them reaching a CR, and seven patients maintained their response (one CR, and six PR). In contrast, four participants in PR progressed late during the maintenance phase (at months 8, 10, 12 and 12, respectively; Table 2). Four patients dropped out during this phase (one for Kaposi sarcoma progression, one for AE/doctor decision, and two for ADR; Fig. 1).

The clinical response was not predicted by Kaposi sarcoma clinical presentation at baseline, including Kaposi sarcoma stage, lesion number or body sites involved, presence of Kaposi sarcoma-associated lymphedema or previous Kaposi sarcoma treatments.

During the post-therapy follow-up, six participants maintained their PR or CR and one in PR showed a further lesion improvement up to CR (Table 2). Two patients lost their response late during this phase (month 10; Table 2).

Of note, improvements in lesion number/nodularity were observed also during the post-therapy follow-up in responders as well as in those progressors that were kept on study after relapse and that did not require systemic Kaposi sarcoma therapy, as the progression was mild or stabilized.

The estimated median duration of response for the participants who entered the maintenance phase was 43 months [95% CI, 28 months–not evaluable (NE)]. The estimated median time to progression was 28 months (95% CI, 18 months–NE).

### Safety evaluation

As per protocol, all 25 enrolled participants were eligible for safety analyses.

No deaths occurred during the study. Seven serious AEs (SAE) were reported, all occurring during the debulking phase (six related to an emergency department admission, one to a cancer diagnosis), and graded as mild (one patient), moderate (five patients), or severe (one patient). Four of these SAE were judged unrelated or unlikely related to study treatment, while three SAE (all of moderate grade) were reported as possibly or probably related to treatment. These included one emergency department admission for paralytic ileus, one for flu-like syndrome, and one for dorsal pain.

The total number of clinical treatment-related non-SAE was 63. Most of them were of mild (59%) or of moderate (40%) grade and occurred (69%) during the induction phase. Only one severe grade AE was recorded (Supplementary Table S2). The most frequent events were gastrointestinal disorders (27 events) and general disorders (22 events; Supplementary Table S3). Two patients withdrew during induction for treatment-related constipation e/o epigastralgia and one during maintenance (epigastralgia).

Most participants (96%) experienced at least one treatment-related laboratory abnormality (Supplementary Table S2). Most of them (69%) occurred during the debulking phase and the vast majority was graded as mild (55%), 38% as moderate, and only 6% as severe (Supplementary Table S2). The most frequent events were hematological abnormalities (151 events), lipid metabolism abnormalities (22 events), reduced renal filtration (14 events), and cylindruria (10 events; Supplementary Table S4). Fifteen events required a temporary reduction and four a temporary discontinuation of treatment. One participant withdrew during induction for indinavir-related crystalluria and one during maintenance for reduced renal filtration.

No significant modifications of the participants’ performance status were observed during the treatment.

### Biological and immunological monitoring

Biological and immunological biomarkers (secondary endpoints) were evaluated in all participants (16) who entered the maintenance phase with indinavir alone, by analyzing the changes from enrolment in responding participants (CR + PR) versus progressors. Baseline levels of these parameters were not statistically different in responders versus progressors.

**Figure 2:**
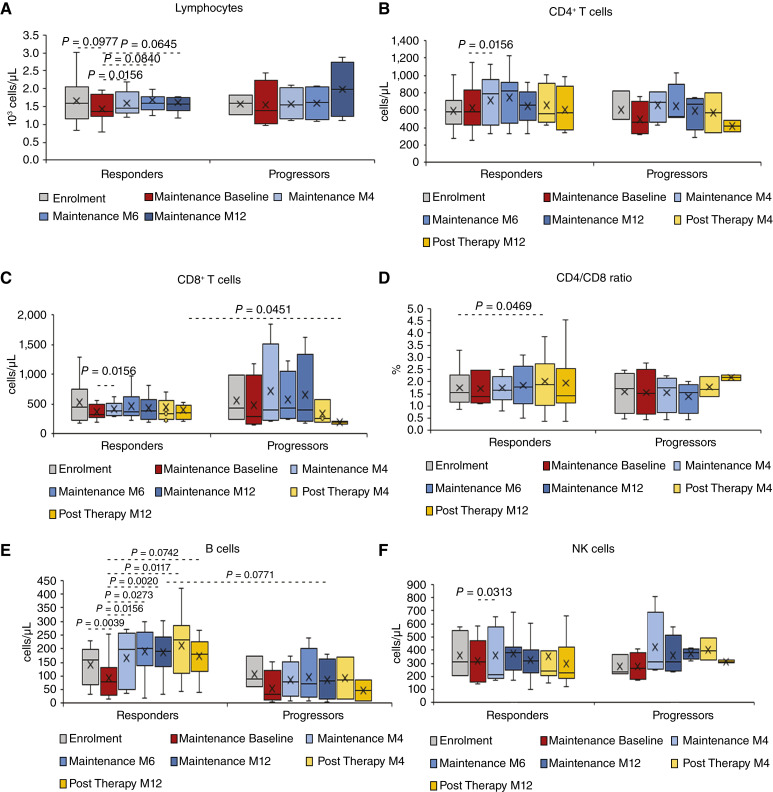
Immunological parameters in participants that entered the maintenance phase (*n* = 16). Levels at enrolment, upon induction and during maintenance and post-therapy follow-up of total lymphocytes absolute number (10^3^ cells/μL; **A**), CD4^+^ (**B**) CD8^+^, and (**C**) T-cell number (cells/μL), CD4^+^/CD8^+^ ratio (**D**), B- (**E**), and NK- (**F**) cell number (cells/μL). Data are presented as box plots. Wilcoxon signed rank sum test for paired data and Mann–Whitney test were used for the analyses. *P* values assess the changes from enrolment and from baseline of maintenance within responders and progressors and differences between responders and progressors at each time point.

As foreseeable, total peripheral blood lymphocytes decreased during the debulking phase, reflecting a chemotherapy-induced myelosuppression, and gradually recovered in the maintenance phase, particularly in responders [maintenance month (M) 4, *P* = 0.0156; M6, *P* = 0.0840; M12, *P* = 0.0645] as compared to maintenance baseline (Fig. 2A).

Responders also showed more relevant changes of lymphocyte subsets than progressors. In particular, CD4^+^ T-cell counts increased in responders reaching a statistically significant difference at M4 of maintenance as compared to their baseline (*P* = 0.0156; Fig. 2B), while they remained stable in progressors. Further, after a decrease upon chemotherapy, responders also showed an increasing recovery of CD8^+^ T-cell counts that reached a statistically significant difference at M4 of maintenance (*P* = 0.0156; Fig. 2C), remaining stable thereafter. Conversely, CD8^+^ T cells showed a gradual reduction in progressors from M6 of maintenance to the end of post-therapy follow-up, resulting significantly lower as compared to responders (*P* = 0.0451; Fig. 2C). As a result, the CD4/CD8 ratio slightly increased in responders, peaking at M4 of post-therapy follow-up as compared to enrolment (*P* = 0.0469; Fig. 2D).

The most relevant result was the significant increase in B-cell levels observed in responders, which was not observed in progressors. In fact, after an initial reduction upon induction with chemotherapy, responders showed a significant recovery of B-cell counts during maintenance that continued over the post-therapy follow-up, as compared to baseline (indinavir alone phase: M4, *P* = 0.0156; M6, *P* = 0.0273; M12, *P* = 0.0020; Post-therapy follow-up: M4, *P* = 0.0117; M12: *P* = 0.0742; Fig. 2E). These levels were substantially higher in responders as compared to progressors over the whole study period.

NK cell levels remained substantially stable in responders except for a reduction at M4 of maintenance as compared to baseline (*P* = 0.0313), whereas a slight and not significant increase was observed in progressors (Fig. 2F).

**Figure 3:**
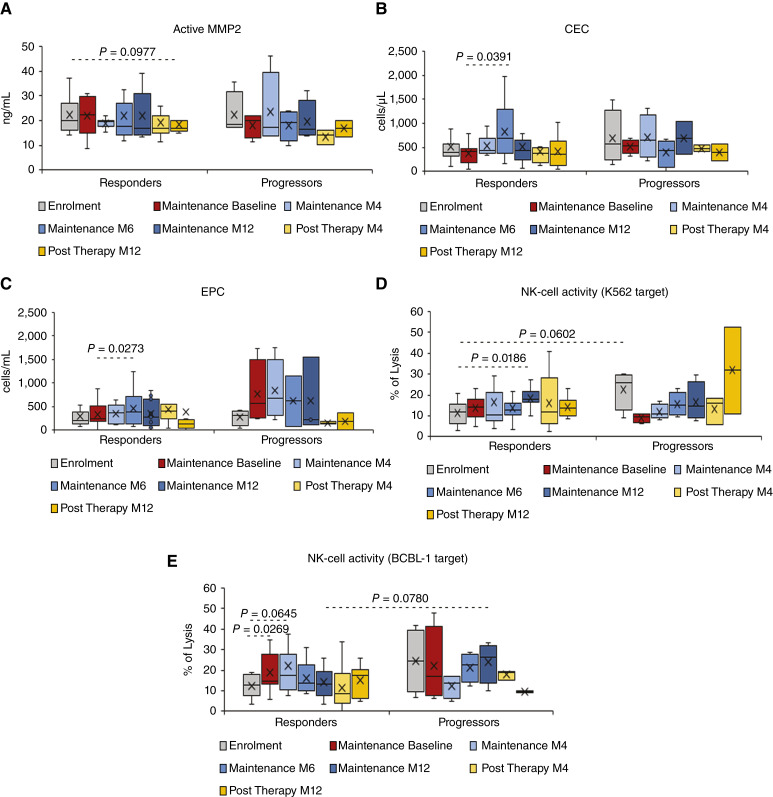
Active MMP-2, CEC, and EPC levels and NK activity in participants that entered the maintenance phase (*n* = 16). Shown are the levels (cells/mL) at enrolment, upon induction, during maintenance, and post-therapy follow-up of active MMP-2 (**A**), CEC (**B**), and EPC (**C**), and NK-cell activity against K562 (**D**) or BCBL-1 targets (**E**) as % of lysis. Data are presented as box plots. Wilcoxon signed rank sum test for paired data and Mann–Whitney test were used for the analyses. *P* values assess the changes from enrolment and from baseline of maintenance within responders and progressors and differences between responders and progressors at each time point.

During the maintenance phase and post-therapy follow-up, responders also showed a gradual decrease of active MMP-2 levels as compared to enrolment, which was not observed in progressors (Fig. 3A).

CEC and EPC levels, which were slightly higher in progressors at enrolment and during treatment, showed an increase during maintenance in both progressors and responders and became significantly higher in responders at M6 of maintenance as compared to their baseline (*P* = 0.0391 and 0.0273, respectively) but not in progressors (Fig. 3B and C).

Finally, lower levels of NK activity against K562 cell targets were observed at enrolment in responders as compared to progressors (Mann–Whitney test, *P* = 0.0602). This activity increased during maintenance particularly in responders, peaking at M12 of maintenance (*P* = 0.0186, as compared to enrolment; Fig. 3D). Responders also showed an increase of NK activity against HHV-8–infected cell targets upon induction (*P* = 0.0269) and at M4 of maintenance (*P* = 0.0645) as compared to enrolment (Fig. 3E), which was not observed in progressors.

## Discussion

The present study was aimed at evaluating the clinical response to a debulking chemotherapy associated with indinavir followed by a maintenance phase with indinavir alone in elderly patients with advanced progressive CKS. All evaluable patients (22) responded to the debulking phase and 16 entered the maintenance phase. Out of these, 12 patients (75%) had a CR or PR and further improvements in lesion number/nodularity were observed upon indinavir alone and during post-therapy follow-up. After relapse, progressors experienced some improvements (including diseased stabilization), did not require systemic Kaposi sarcoma therapy, and were kept on study. These data suggest that indinavir exerts a long-term clinical benefit in patients with a progressive disease by boosting and maintaining the effect of chemotherapy.

Although clinical studies on CKS are scarce and difficult to compare because of the rarity of the disease and the lack of a standardized staging and clinical outcome evaluation (4), the response rate observed upon induction with the debulking chemotherapy and indinavir combined appears not inferior to what was observed in previous (mostly retrospective) studies based on vinblastine, vinblastine plus bleomycin or paclitaxel (18–22) or observed with other conventional chemotherapies (4). In particular, the overall response rate previously achieved in the same clinical center with vinblastine plus bleomycin was virtually 100% (97%; ref. 18), it was lower with vinblastine alone and paclitaxel (58% and 82%, respectively; refs. 19, 20), whereas in our study, the combined strategy with indinavir raised efficacy to 100%. Similarly, more recent approaches like pembrolizumab (15) or pomalidomide (16) reached only 75% and 80% of response rate, respectively.

Further, our study indicates an estimated median duration of clinical response of 43 months (95% CI, 28 months–not evaluable), which is better than that previously reported in historical data from the same clinical center with chemotherapy alone. In particular, the median duration of response reported for vinblastine alone was 20+ or 6.5 for patients in CR or PR, respectively (calculated from time of best response; ref. 19), and of 4+ months for vinblastine + bleomycin (1–25+, calculated after therapy stop; ref. 18), while our median duration of response calculated from therapy stop is 27 months (IQR 18), and 25 months from best response, respectively. Our results are also better as compared to pembrolizumab or pomalidomide (estimated median duration of 23 and 6 months, respectively; refs. 15, 16). Thus, the results of this study suggest that the addition of HIV-PI to chemotherapy is effective in increasing the duration of clinical response to chemotherapy in patients with progressive advanced CKS.

Overall, the treatment was well tolerated as most of the AEs were expected in an elderly population presenting with preexisting and concurrent diseases or laboratory abnormalities at enrolment, thus confirming the safety already observed in the previous trial (12). In particular, no deaths occurred during the study and the only three SAEs possibly or probably related to treatment were of moderate grade. Similarly, most clinical or laboratory treatment-related AEs were of mild or moderate grade (98% and 93%, respectively) and mostly occurred with chemotherapy.

Monitoring of immunological and biological markers associated with Kaposi sarcoma pathogenesis or HIV-PI activity also showed an improvement in responders as compared to progressors.

In particular, an amelioration of the immune status with an increase of total lymphocytes, CD4^+^ and CD8^+^ T cells, and CD4/CD8 ratio was observed in responders as compared to progressors; however, the most striking change was the increase in B-cell levels observed in responders during the maintenance phase with indinavir alone and post-therapy follow-up, which was not observed in progressors. Of note, previous data indicated that higher B-cell counts are associated with a reduced risk of Kaposi sarcoma development and with increased response rates upon therapy (23–25). As B cells are the target of HHV-8 (26), their recovery may be suggestive of HHV-8 infection control by the host. On the other hand, B cells, through secretion of both neutralizing and non-neutralizing virus-specific antibodies, are a key component of immune control of virus infections and tumor surveillance (27). In addition, evidence also indicates that B cells may have antitumor activity, which is mediated by killing of tumor cells through expression of TNFs/receptor proteins or production of granzyme B (28), IL12-mediated activation of NK cells (29), or antibody-dependent cell-mediated cytotoxicity (30).

Among the parameters associated with tumor angiogenesis and cell invasion, only responders showed a decrease of active MMP-2 levels during maintenance with indinavir and post-therapy follow-up. This result is of particular interest as MMP-2 activity is required for angiogenesis, tumor cell invasion, and spread (2) and MMPs are a target of HIV-PI activity in Kaposi sarcoma and other tumor models (6–11).

Interestingly, we also found that CEC and EPC levels were low in responders as compared to progressors both at enrolment and during treatment. Rare in healthy individuals, CEC are shed from vessel walls and enter the circulation reflecting EC damage or dysfunction due to immunoactivation, or as found in patients with cancer, may correlate with tumor progression (31). Of note, a reduction in CEC was observed in patients with early CKS responding to indinavir treatment (12). Similarly, EPC were detected at increased frequency in the circulation of patients with CKS and may represent potential HHV-8 reservoirs and putative precursors of Kaposi sarcoma spindle cells (32).

Finally, responders showed improvements of NK-cell activity, a property of cytotoxic innate lymphoid cells that is involved in the elimination of virus-infected cells and tumors and which is reduced in patients with Kaposi sarcoma (33, 34). Noticeably, HHV-8 is known to evade cytotoxic T-cell responses by downregulating MHC class I molecules, while inhibiting the ensuing NK-cell response by downregulating NK co-activation molecules (35, 36). Hence, these data may in part explain the inhibition of HHV-8 replication/shedding selectively associated with HIV-PI-based antiretroviral therapy (37).

The main limitations of this study are the small number of participants, the lack of a control group, the need to rely on historical data for comparisons of response rates and response duration. However, Kaposi sarcoma is a rare disease and, therefore, has a low incidence and, in addition, patients with advanced Kaposi sarcoma are elderly with frequent concomitant pathologies and treatments, which may not meet the inclusion criteria of a clinical trial. However, this is a common limitation encountered by all studies evaluating the clinical efficacy of anti-Kaposi sarcoma treatments and indeed the number of participants of our trial is in line with the other studies conducted in CKS.

Overall, our results support the anti-Kaposi sarcoma activity of HIV-PIs and further suggest that in advanced CKS a combination therapy including indinavir with vinblastine ± bleomycin or taxol is well tolerated and, while achieving comparable efficacy, is capable of extending the duration of clinical responses as compared to conventional chemotherapy.

In this context, we have recently shown that HIV-PIs reduce tumor hypoxia and enhance the delivery and antitumor activity of conventional chemotherapy in a preclinical model of cervical cancer (9). Furthermore, since HIV-PI sensitize tumor cells to the apoptotic effects of cytotoxic agents (5, 6), indinavir may potentiate the antitumor actions of vinblastine and bleomycin. Moreover, our results indicate that treatment is associated with amelioration of the immune status, suggesting that HIV-PI treatment may also promote a better control of HHV-8 infection and improve tumor-cell killing.

Other HIV-PIs like nelfinavir or lopinavir are also being tested in other tumor settings. In particular, a phase-2 clinical trial showed that use of lopinavir monotherapy is safe and effective in cervical intraepithelial-neoplasia lesions developing in HIV-negative women (38). Based on their radio- and chemo-sensitization properties showed in preclinical studies, HIV-PIs are also being clinically tested in combination with radiotherapy or conventional chemotherapy for the treatment of other advanced/refractory solid tumors, including myeloma, prostate, pancreatic, lung, or cervical cancers, with promising results (39).

The therapeutic strategy shown in our study may represent an important treatment improvement for elderly patients with advanced, progressive CKS. As most HIV-PIs are orally available drugs with a well-defined pharmacodynamics, are already available as generic drugs, and are less expensive than more recent approaches based on monoclonal antibodies or new compounds, the repositioning of HIV-PIs for Kaposi sarcoma as well as other tumor treatments may have a strong, beneficial impact on patients’ clinical management and quality of life and on the costs for the National Health Systems.

## Supplementary Material

Table S1 Supplementary Table 1 shows the active concurrent illnesses at baseline.

Table S2 Supplementary Table 2 shows the treatment-related AE numbers by study phases.

Table S3 Supplementary Table 3 shows the clinical treatment-related AEs by SOC and study phases.

Table S4 Supplementary Table 4 shows the treatment-related laboratory AE by SOC, preferred terms and study phases.

Table S5 Supplementary Table 5 summarizes the representativeness of study participants
