# Supplementary material for: Clinical Efficacy of the HIV Protease Inhibitor Indinavir in Combination with Chemotherapy for Advanced Classic Kaposi Sarcoma Treatment: A Single-Arm, Phase II Trial in the Elderly
Source: Cancer Res Commun. 2024 Aug 15;4(8):2112–22. doi: 10.1158/2767-9764.CRC-24-0102 (PMC11324028; doi:10.1158/2767-9764.CRC-24-0102)
Supplement: Table S1 — Supplementary Table 1 shows the active concurrent illnesses at baseline. [file crc-24-0102_table_s1_suppst1.docx]

Supplementary Table 1. Active concurrent illnesses at baseline by MedDRA system organ class.

| **Active concurrent illnesses** | **Treatment groups** | | |
| --- | --- | --- | --- |
|  | **Vinblastine/**  **bleomycin+**  **indinavir** | **Vinblastine**  **alone**  **+indinavir** | **Total** |
|  | n (%) | n (%) | n (%) |
| **Cardiovascular** | 3 (20%) | 6 (60%) | **9 (36%)** |
| **Respiratory** | 1 (7%) | 2 (20%) | **3 (12%)** |
| **Gastrointestinal** | 5 (33%) | 1 (10%) | **6 (24%)** |
| **Urological** | 4 (27%) | 3 (30%) | **7 (28%)** |
| **Endocrine-Metabolic** | 7 (47%) | 2 (20%) | **9 (36%)** |
| **Hematologic-Lymphatic** | 1 (7%) | 0 (0%) | **1 (4%)** |
| **Dermatologic** | 1 (7%) | 1 (10%) | **2 (8%)** |
| **Musculoskeletal** | 2 (13%) | 0 (0%) | **2 (8%)** |
| **Neurological** | 1 (7%) | 1 (10%) | **2 (8%)** |
| **Other** | 0 (0%) | 2 (20%) | **2 (8%)** |

Data are shown as absolute number and percentage (%)
