# Supplementary material for: Clinical Efficacy of the HIV Protease Inhibitor Indinavir in Combination with Chemotherapy for Advanced Classic Kaposi Sarcoma Treatment: A Single-Arm, Phase II Trial in the Elderly
Source: Cancer Res Commun. 2024 Aug 15;4(8):2112–22. doi: 10.1158/2767-9764.CRC-24-0102 (PMC11324028; doi:10.1158/2767-9764.CRC-24-0102)
Supplement: Table S2 — Supplementary Table 2 shows the treatment-related AE numbers by study phases. [file crc-24-0102_table_s2_suppst2.docx]

**Supplementary Table 2.** **Treatment-related adverse events.** The table shows the number of participants experiencing at least one treatment-related (certain, probable, and possible relationship) clinical or laboratory adverse event and the total number of events reported by treatment phase (Safety population) (total N and N/treatment phase).

|  | **Treatment phase** | | **Total**  **(N = 25)** |
| --- | --- | --- | --- |
|  | **Induction**  **(N = 25)** | **Maintenance**  **(N = 16)** |  |
| **Clinical adverse events** |  |  |  |
| Number of subjects with at least 1 event | 17 (68%) | 4 (25%) | **18 (72%)** |
| Total no. of events reported | 50 (79%) | 13 (21%) | **63 (100%)** |
| Intensity |  |  |  |
| Mild | 24 (48%) | 10 (77%) | **34 (54%)** |
| Moderate | 25 (50%) | 3 (23%) | **28 (44%)** |
| Severe | 1 (2%) | 0 (0%) | **1 (2%)** |
| **Laboratory abnormalities** |  |  |  |
| Number of subjects with at least 1 event | 24 (96%) | 16 (100%) | **24 (96%)** |
| Total n. of events | 242 (69%) | 108 (31%) | **350 (100%)** |
| Intensity |  |  |  |
| Mild | 209 (86%) | 89 (84%) | **194 (55%)** |
| Moderate | 23 (10%) | 11 (10%) | **134 (38%)** |
| Severe | 10 (4%) | 8 (7%) | **22 (6%)** |

Data are shown as absolute number and percentage (%)
