# Supplementary material for: Clinical Efficacy of the HIV Protease Inhibitor Indinavir in Combination with Chemotherapy for Advanced Classic Kaposi Sarcoma Treatment: A Single-Arm, Phase II Trial in the Elderly
Source: Cancer Res Commun. 2024 Aug 15;4(8):2112–22. doi: 10.1158/2767-9764.CRC-24-0102 (PMC11324028; doi:10.1158/2767-9764.CRC-24-0102)
Supplement: Table S3 — Supplementary Table 3 shows the clinical treatment-related AEs by SOC and study phases. [file crc-24-0102_table_s3_suppst3.docx]

Supplementary Table 3. Clinical treatment-related adverse events by system organ classes, preferred terms and study phases (safety population).

|  |  | **Treatment phase** | |  |
| --- | --- | --- | --- | --- |
| **SOC** | **Preferred Term** | **Induction**  n (%) | **Maintenance**  n (%) | **Total**  n (%) |
| **GASTROINTESTINAL DISORDERS** | epigastric discomfort | 8 (30%) | 2 (7%) | 10 (37%) |
|  | nausea | 4 (15%) | 0 (0%) | 4 (15%) |
|  | constipation | 3 (11%) | 0 (0%) | 3 (11%) |
|  | abdominal pain | 2 (7%) | 0 (0%) | 2 (7%) |
|  | abdominal pain and constipation | 1 (4%) | 0 (0%) | 1 (4%) |
|  | dyspepsia and nausea | 1 (4%) | 0 (0%) | 1 (4%) |
|  | epigastric and esophageal discomfort | 1 (4%) | 0 (0%) | 1 (4%) |
|  | gastritis | 1 (4%) | 0 (0%) | 1 (4%) |
|  | nausea and vomiting | 1 (4%) | 0 (0%) | 1 (4%) |
|  | stomach discomfort | 1 (4%) | 0 (0%) | 1 (4%) |
|  | **Total** | 24 (89%) | 3 (11%) | 27 (100%) |
| **GENERAL DISORDERS** | asthenia | 9 (41%) | 0 (0%) | 9 (41%) |
| **ADMINISTRATION SITE CONDITIONS** | pyrexia | 8 (36%) | 0 (0%) | 8 (36%) |
|  | drug fever | 3 (14%) | 0 (0%) | 3 (14%) |
|  | Influenza like illness | 1 (5%) | 0 (0%) | 1 (5%) |
|  | pyrexia and asthenia | 1 (5%) | 0 (0%) | 1 (5%) |
|  | **Total** | 22 (100%) | 0 (0%) | 22 (100%) |
| **IMMUNE SYSTEM DISORDERS** | allergy to chemicals | 0 (0%) | 1 (100%) | 1 (100%) |
|  | **Total** | 0 (0%) | 1 (100%) | 1 (100%) |
| **MUSCULOSKELETAL AND CONNECTIVE** | musculoskeletal pain | 2 (67%) | 0 (0%) | 2 (67%) |
| **TISSUE DISORDERS** | myalgia | 1 (33%) | 0 (0%) | 1 (33%) |
|  | **Total** | 3 (100%) | 0 (0%) | 3 (100%) |
| **SKIN AND SUBCUTANEOUS TISSUE** | dermatitis and eczema | 0 (0%) | 4 (36%) | 4 (36%) |
| **DISORDERS** | dermatitis allergic | 0 (0%) | 2 (18%) | 2 (18%) |
|  | lichenoid dermatitis | 0 (0%) | 2 (18%) | 2 (18%) |
|  | drug eruption | 0 (0%) | 1 (9%) | 1 (9%) |
|  | eczema | 0 (0%) | 1 (9%) | 1 (9%) |
|  | palmar and plantar erythema | 1 (9%) | 0 (0%) | 1 (9%) |
|  | **Total** | 1 (9%) | 10 (91%) | 11 (100%) |

Data are shown as absolute number and percentage (%)
