# Supplementary material for: Clinical Efficacy of the HIV Protease Inhibitor Indinavir in Combination with Chemotherapy for Advanced Classic Kaposi Sarcoma Treatment: A Single-Arm, Phase II Trial in the Elderly
Source: Cancer Res Commun. 2024 Aug 15;4(8):2112–22. doi: 10.1158/2767-9764.CRC-24-0102 (PMC11324028; doi:10.1158/2767-9764.CRC-24-0102)
Supplement: Table S4 — Supplementary Table 4 shows the treatment-related laboratory AE by SOC, preferred terms and study phases. [file crc-24-0102_table_s4_suppst4.docx]

**Supplementary Table 4. Treatment-related laboratory adverse events by system organ class, preferred terms and study phase (safety population)**

|  |  | **Treatment phase** | |  |
| --- | --- | --- | --- | --- |
|  |  | **Induction**  n (%) | **Maintenance**  n (%) | **Total**  n (%) |
| **CHEMISTRY** | LDL cholesterol increased | 15 (10%) | 7 (5%) | 22 (15%) |
|  | total cholesterol increased | 14 (9%) | 6 (4%) | 20 (13%) |
|  | triglycerides increased | 12 (8%) | 6 (4%) | 18 (12%) |
|  | HDL cholesterol decreased | 10 (7%) | 4 (3%) | 14 (9%) |
|  | creatinine clearance decreased | 7 (5%) | 7 (5%) | 14 (9%) |
|  | indirect bilirubin increased | 4 (3%) | 4 (3%) | 8 (5%) |
|  | amylase increased | 3 (2%) | 4 (3%) | 7 (5%) |
|  | creatinine increased | 4 (3%) | 3 (2%) | 7 (5%) |
|  | total bilirubin increased | 3 (2%) | 4 (3%) | 7 (5%) |
|  | gamma GT increased | 4 (3%) | 2 (1%) | 6 (4%) |
|  | glucose increased | 4 (3%) | 2 (1%) | 6 (4%) |
|  | ALT increased | 1 (1%) | 3 (2%) | 4 (3%) |
|  | blood urea nitrogen increased | 1 (1%) | 3 (2%) | 4 (3%) |
|  | uric acid increased | 3 (2%) | 1 (1%) | 4 (3%) |
|  | AST increased | 1 (1%) | 2 (1%) | 3 (2%) |
|  | Alkaline phosphatase increased | 2 (1%) | 0 (0%) | 2 (1%) |
|  | CPK increased | 2 (1%) | 0 (0%) | 2 (1%) |
|  | albumin decreased | 0 (0%) | 1 (2%) | 1 (2%) |
|  | direct bilirubin increased | 1 (1%) | 0 (0%) | 1 (2%) |
|  | total protein decreased | 1 (2%) | 0 (0%) | 1 (2%) |
|  | **Total** | 92 (61%) | 59 (39%) | 151 (100) |
| **ELECTROLYTES** | Ca++ decreased | 1 (10%) | 4 (40%) | 5 (50%) |
|  | K increased | 1 (10%) | 2 (20%) | 3 (30%) |
|  | Na decreased | 1 (10%) | 1 (10%) | 2 (20%) |
|  | **Total** | 3 (30%) | 7 (70%) | 10 (100) |
| **HEMATOLOGY** | leucocytes decreased | 30 (20%) | 4 (3%) | 34 (23%) |
|  | hematocrit decreased | 19 (13%) | 5 (3%) | 24 (16%) |
|  | hemoglobin decreased | 18 (12%) | 4 (3%) | 22 (15%) |
|  | RBC decreased | 16 (11%) | 6 (4%) | 22 (15%) |
|  | lymphocytes decreased | 12 (8%) | 4 (3%) | 16 (11%) |
|  | neutrophils decreased | 13 (9%) | 1 (1%) | 14 (9%) |
|  | monocytes decreased | 10 (7%) | 0 (0%) | 10 (7%) |
|  | platelets decreased | 5 (3%) | 0 (0%) | 5 (3%) |
|  | eosinophils decreased | 2 (1%) | 0 (0%) | 2 (1%) |
|  | leucocytes increased | 1 (1%) | 0 (0%) | 1 (1%) |
|  | neutrophils increased | 1 (1%) | 0 (0%) | 1 (1%) |
|  | **Total** | 127 (84%) | 24 (16%) | 151 (100) |
| **URINALYSIS** | cylindruria hyaline and/or hayline-granular | 6 (16%) | 4 (11%) | 10 (26%) |
|  | leukocyturia | 3 (8%) | 4 (11%) | 7 (18%) |
|  | indinavir crystalluria | 4 (11%) | 2 (5%) | 6 (16%) |
|  | cylindruria hyaline-granular and erytrocytic | 3 (8%) | 2 (5%) | 5 (13%) |
|  | hematuria | 1 (3%) | 1 (3%) | 2 (5%) |
|  | micro hematuria. glomerular | 0 (0%) | 2 (5%) | 2 (5%) |
|  | proteinuria | 1 (3%) | 1 (3%) | 2 (5%) |
|  | albuminuria | 0 (0%) | 1 (3%) | 1 (3%) |
|  | cylindruria hyaline-granular and leukocytic | 1 (3%) | 0 (0%) | 1 (3%) |
|  | glomerular micro-hematuria | 0 (0%) | 1 (3%) | 1 (3%) |
|  | micro albuminuria | 1 (3%) | 0 (0%) | 1 (3%) |
|  | **Total** | 20 (53%) | 18 (47%) | 38 (100% |

Data are shown as absolute number and percentage (%)
