# Supplementary material for: Clinical Efficacy of the HIV Protease Inhibitor Indinavir in Combination with Chemotherapy for Advanced Classic Kaposi Sarcoma Treatment: A Single-Arm, Phase II Trial in the Elderly
Source: Cancer Res Commun. 2024 Aug 15;4(8):2112–22. doi: 10.1158/2767-9764.CRC-24-0102 (PMC11324028; doi:10.1158/2767-9764.CRC-24-0102)
Supplement: Table S5 — Supplementary Table 5 summarizes the representativeness of study participants [file crc-24-0102_table_s5_suppst5.docx]

| **Supplementary Table 5.** Representativeness of Study Participants | |
| --- | --- |
| Cancer type(s)/subtype(s)/stage(s)/condition | **Classic Kaposi’s sarcoma** (KS) is a rare angio-proliferative tumor with a relatively indolent course that occurs in elderly people of Mediterranean, Eastern European and Middle Eastern heritage.  Several other clinical-epidemiological forms have been described, including iatrogenic KS (developing in patients treated with chronic immunosuppressive therapy, especially in organ transplant recipients), endemic KS (an aggressive form involving visceral and/or lymphatic organs occurring in children and young adults from sub-equatorial Africa), and epidemic/HIV-associated KS (characterized by a particularly aggressive course and representing one of the most frequent tumor in HIV-infected patients, even after the introduction of effective antiretroviral regimens).  All KS forms are associated with infection by the human herpesvirus 8 (HHV8) and share the same histopathology. |
| Considerations related to: | |
| Sex | All KS forms, including classic KS, occur more frequently in men (male/female ratio 4:1). |
| Age | Classic KS incidence exponentially increases with age (median age at the time of diagnosis between the sixth and the seventh decade of life). |
| Race/ethnicity/geography | Although a higher incidence of classic KS has been reported to occur in Mediterranean populations and in certain ethnic groups of Ashkenazi or Shepardnazi Jewish descent, this pattern seems more to reflects geographic characteristics than ethnicity. In particular, the overall KS incidence correlates with HHV8 seroprevalence in the general population, which differs by region and age group.  The incidence rates of classic KS in European population-based registries are markedly variable. Low rates were reported in UK (0.14 per million in both males and females between 1971–1980), whereas higher rates were reported in Italy (10.5 per million in men and 2.7 per million in women, between 1976–1984), with the highest incidence rates reported for 2 Mediterranean Italian islands: Sardinia (24.3 per million in men and 7.7 per million in women, between 1977–1991) and Sicily (30.1 per million in men and 5.4 per million in women, between 1976–1984). |
| Overall representativeness of this study | Our study focused to classic KS participants to avoid the confounding of the immune recovery promoted by the anti-HIV-activity of Indinavir.  The age and sex distribution of participants in our study reflects the ones reported in the literature by epidemiological studies. Of note, the baseline characteristics of our patient population are comparable to those of other therapeutic trial conducted in advanced classic KS.  Early-stage classic KS patients were not included in the study since data obtained in our previous trial indicate that in these patients a clinical response may be obtained with Indinavir alone, and that chemotherapy can be avoided to spare them toxicity  Our study population was limited to one clinical center in Italy. This allowed a comparison with historical data obtained in the same center with vinblastine/bleomycin alone. |
